# Oral Supplementation of *Houttuynia cordata* Extract Reduces Viremia in PRRSV-1 Modified-Live Virus-Vaccinated Pigs in Response to the HP-PRRSV-2 Challenge

**DOI:** 10.3389/fimmu.2022.929338

**Published:** 2022-07-18

**Authors:** Wilawan Ruansit, Wasin Charerntantanakul

**Affiliations:** Research laboratory for immunity enhancement in humans and domestic animals, Program of Biotechnology, Faculty of Science, Maejo University, Chiang Mai, Thailand

**Keywords:** porcine reproductive and respiratory syndrome virus, *houttuynia cordata*, modified-live virus vaccine, interferon, cross-protection

## Abstract

This study evaluated the *in vitro* antiviral activities and the *ex vivo* immunomodulatory effects of *Houttuynia cordata* Thunb. (HC) ethanolic extracts in response to porcine reproductive and respiratory syndrome virus (PRRSV). In addition, this study evaluated the *in vivo* effects of oral supplementation of HC extract on immune responses to and cross-protective efficacy of PRRSV-1 modified-live virus (MLV) vaccine against the highly pathogenic (HP)-PRRSV-2 challenge. *In vitro* experiments demonstrated that HC extracted in either 50%, 70%, or 95% ethanol (referred to as HC50, HC70, and HC95, respectively) significantly interfered with PRRSV replication in MARC-145 cells. *Ex vivo* experiments revealed that all HC extracts significantly enhanced mRNA expressions of type I interferon-regulated genes, type I and II interferon (IFN), and pro- and anti-inflammatory cytokines in HP-PRRSV-2-inoculated monocyte-derived macrophages. An *in vivo* experiment included four groups of six pigs (4 weeks old; *n* = 24). Group 1 and group 2 were vaccinated with the PRRSV-1 MLV vaccine at 0 dpv (day post vaccination). Group 2 also received oral administration of HC50 extract at 0–49 dpv. Group 3 received the PRRSV-1 MLV vaccine solvent at 0 dpv, while group 4 served as strict control. Groups 1–3 were challenged intranasally with HP-PRRSV-2 at 28 dpv and immune-related and clinical parameters were monitored weekly until 49 dpv. Compared to group 1, group 2 demonstrated significantly increased IFN regulatory factor 3 mRNA expression of PRRSV-recalled peripheral blood mononuclear cells, and significantly reduced HP-PRRSV-2 viremia. No difference in PRRSV-specific antibody responses, rectal temperature, clinical scores, and average daily weight gain was detected. Our study reports the immunomodulatory and anti-PRRSV potentials of HC extract in PRRSV-1 MLV-vaccinated/HP-PRRSV-2 challenged pigs.

## 1 Introduction

Porcine reproductive and respiratory syndrome virus (PRRSV) causes severe economic losses in the swine industry worldwide. The virus causes reproductive failures in breeding swine and respiratory distress, poor growth performance, and mortality in growing pigs.

PRRSV belongs to the genus *Porarterivirus*, family *Arteriviridae*. The virus has a positive-sense single-stranded RNA genome with an envelope. It can be divided into two species, i.e., PRRSV-1 and PRRSV-2, based on its 3**’**-terminal structural genes (1). Both PRRSV species share approximately 60% nucleotide sequence homology to each other (2). In each PRRSV species, there exist classical PRRSV strains that cause mild to severe losses, and highly pathogenic PRRSV **(**HP-PRRSV**)** strains that often cause severe losses (3).

PRRSV infects and replicates in myeloid antigen (Ag)-presenting cells (APCs), i.e., monocytes, macrophages, and dendritic cells (4). Following infection, PRRSV poorly induces innate immune responses, e.g., phagocytic and microbicidal activities, Ag presentation and T-cell activation, expression of pattern recognition receptors [e.g., Toll-like receptor 3 **(**TLR3**)** and TLR7], production of type I and II interferons **(**IFN**)**, and production of pro-inflammatory cytokines [e.g., interleukin-1 **(**IL-1**)** and tumor necrosis factor alpha **(**TNFα**)**] (5–7). Poor induction of innate immune activities by PRRSV has been reported to be attributed to several PRRSV proteins, i.e., non-structural protein 1 **(**nsp1**)** (8), nsp2 (9), nsp4 (10), nsp5 (11), nsp11 (12), glycoprotein 5 (13), and nucleocapsid (N) proteins (14). These viral proteins negatively interfere with innate immune responses *via* several mechanisms, which include suppression of signaling molecule and transcription factor activation [e.g., extracellular signal-regulated kinase (15), retinoic acid-induced gene-1 (12), mitochondrial antiviral signaling protein (12), IFN regulatory factor 3 **(**IRF3**)** (8), NFκB (16), and STAT1 (11)], virus-mediated degradation of CREB-binding protein (17), and virus inhibition of nuclear translocation of STAT1 and STAT2 (11).

In contrast to a poor induction of pro-inflammatory innate immune activities, PRRSV significantly promotes anti-inflammatory cytokine expressions, i.e., IL-1 receptor antagonist (IL-1Ra), IL-10, and transforming growth factor beta **(**TGFβ**)** of infected myeloid APCs, T helper 2 (Th2) cells, and regulatory T cells (5, 6, 18, 19). Weak pro-inflammatory innate immune activities of PRRSV-infected myeloid APCs and a rather robust anti-inflammatory response contribute to a relatively weak and delayed PRRSV-specific adaptive cell-mediated immune (CMI) response, particularly cytotoxic T lymphocytes (CTLs) and Th1 cells (7). These patterns of immune responses are seen more distinctively following classical PRRSV infection than HP-PRRSV infection. The immune responses to HP-PRRSV are relatively faster and stronger, particularly production of pro-inflammatory cytokines (20).

Current protocols for PRRSV control include vaccination. A vaccine produced from the same virus species as infecting PRRSV is recommended as it confers better clinical protection than a vaccine generated from different PRRSV species (21). In many countries, including Thailand, a co-infection of PRRSV-1 and PRRSV-2 does exist, and vaccine’s limited cross-protective efficacy often results in clinical losses (22). An ideal PRRSV vaccine should elicit good innate and adaptive CTLs and Th1 responses, and confer good cross-protection (21). Numerous efforts have been made to enhance PRRSV vaccine immunogenicity and cross-protective efficacy (21, 23–28). Several forms of PRRSV vaccines, both traditional and innovative, have been generated and experimented with various types of vaccine adjuvants. As of present, PRRSV modified-live virus (MLV) vaccine and killed virus vaccine adjuvanted with some vaccine adjuvants, i.e., CpG oligodeoxynucleotides, Montanide^TM^ Gel 01 ST, poly**(**lactic-co-glycolic**)** acid, and peptide nanofiber hydrogel, showed improved adaptive CMI response and cross-protection (23–26). These studies suggest that a potential adjuvant or immunomodulator is essential for PRRSV vaccine.

*Houttuynia cordata* Thunb. (HC) is a medicinal plant indigenous to countries in East and Southeast Asia including Thailand. The plant possesses many biological properties including immunomodulation, antivirus, and anticancer (29). HC reportedly induced a balanced pro- and anti-inflammatory immune response (30–34). Treatment with HC extract in murine macrophages stimulated with lipopolysaccharide (LPS) and in human mast cell line stimulated with phorbol myristate acetate plus a calcium ionophore reduced expressions of several inflammatory markers, e.g., inducible nitric oxide synthase, nitric oxide, cyclooxygenase-2, prostaglandin E2, IL-1β, IL-6, and TNFα (31, 32, 34). The HC treatment also inhibited NFκB phosphorylation, nuclear translocation, and MAPK activation in those cells (31, 32, 34). In addition, treatment with HC extract stimulated the proliferation of mouse spleen lymphocytes and their secretion of IL-2 (30). In an *in vivo* study, oral supplementation of HC extract enhanced IFNβ secretion and reduced TNFα secretion in the lung of influenza virus H1N1-infected mice, which significantly attenuated virus-mediated acute lung injury (33). HC contains bioactive flavonoids, e.g., rutin, quercetin, quercitrin, isoquercitrin, and houttuynoids (35, 36). Some of these flavonoids, i.e., rutin and quercetin, have been reported to inhibit PRRSV replication in MARC-145 cells and induce IFN-regulated genes (IRGs) and type I and II IFN gene expressions in porcine monocyte-derived macrophages (MDMs) (37, 38). The immunomodulatory and antiviral properties of HC have not been evaluated in pigs in either *ex vivo* or *in vivo* settings. Understanding of such properties of HC may lead to the exploitation of HC for PRRSV control.

The objectives of this study were to evaluate the *ex vivo* immunomodulatory effects and *in vitro* anti-PRRSV effects of HC extracts, and the *in vivo* effects of oral supplementation of HC extract on immune response to the PRRSV-1 MLV vaccine and cross-protection against the HP-PRRSV-2 challenge in pigs.

## 2 Materials and Methods

### 2.1 PRRSV

PRRSV-1 VP046 Bis strain (Amervac^®^ PRRS MLV, Batch 97PT-6; Hipra, Spain) and Thai HP-PRRSV-2 isolate 10PL01 [lineage 8 (39)] were propagated in MARC-145 cells in MEM^++^ [MEM (Caisson Laboratories, Smithfield, UT], 10% heat-inactivated FBS (Capricorn Scientific GmbH, Germany), and 1% antibiotics/antimycotic (Gibco, Grand Island, NY). Their titers were determined by immunoperoxidase monolayer assay (IPMA), using mouse mAb specific for PRRSV N proteins (IgG2b, clone 5C61; Median Diagnostics, South Korea) and HRP-conjugated goat anti-mouse IgG (CiteAB, UK). PRRSV-1 and HP-PRRSV-2 at their fifth and tenth passage, respectively, in MARC-145 cells were used. Mock Ags were prepared in the same fashion as virus Ags, but with the absence of PRRSV.

### 2.2 Animals

Crossbred pigs (Large White/Landrace x Duroc) seronegative to PRRSV and *Mycoplasma hyopneumoniae* as determined by ELISA (IDEXX Laboratories, Westbrook, ME) were used for *ex vivo* experiments (8 weeks old; *n* = 4) and an *in vivo* experiment (4 weeks old; *n* = 24). They were housed at the swine research extension facility (Chiang Mai, Thailand), monitored daily, and fed *ad libitum* with commercial feed.

### 2.3 Preparation of HC Extracts

Fresh aerial parts of HC were collected from farms in Chiang Mai, Thailand during June–August, 2017. They were rinsed several times with tap water, then air-dried **(**24 h**)**, oven-dried **(**50^o^C, 72 h**)**, and pulverized. The pulverized HC yielded approximately 10**%** of fresh HC weight. Three concentrations of ethanol (50%, 70%, and 95%) were used for extraction at 10**%** w/v. The suspensions were stirred (150 rpm, 72 h), and centrifuged **(**2,800 ×*g*, 4^o^C, 10 min**).** The supernatants were filtered through grade 1 filter paper **(**Whatman^®^, Merck, Germany**).** The filtrates were subjected to rotary evaporation **(**40^o^C**) (**Buchi^®^, New Castle, DE), followed by lyophilization **(**Martin Christ, Germany**).** The extract powder **(**referred to as HC50, HC70, and HC95; each yielded approximately 10**%** of pulverized HC weight**)** was aliquoted, stored at −20^o^C, and protected from light until use. When used for *in vitro* and *ex vivo* experiments, all HC extracts were resuspended with dimethyl sulfoxide **(**DMSO; Sigma, St. Louis, MO**)** and then placed into the Detoxi-gel^TM^ endotoxin removal columns **(**Thermo Fisher Scientific Inc., Waltham, MA**).** The eluates were subsequently filtered through a 0.22-µm filter (Minisart^®^, Sartorius, France). The presence of LPS in all eluates was less than 0.25 EU/ml as determined by limulus amebocyte lysate assay (Chromo LAL^®^, Associates of Cape Cod, Inc., East Falmouth, MA). Filtered eluates were diluted with MEM^++^ before use for *in vitro* experiments in MARC-145 cells, or with RPMI^++^ (RPMI-1640 with L-glutamine (Caisson Laboratories), 10% heat-inactivated FBS, and 1% antibiotics/antimycotic) before use for *ex vivo* experiments in MDMs. When used for oral administration *in vivo*, the HC50 extract was resuspended in DMSO and further diluted with sterile water. Vehicle control was prepared in the same fashion as the filtered eluates (for *in vitro* and *ex vivo* experiments) and HC50 extract (for *in vivo* experiment) except that no HC was included. The voucher specimens of HC have been deposited at the Faculty of Science, MJU.

### 2.4 High-Performance Liquid Chromatography

All HC extracts (1.0 g each) were resuspended with 2N NaOH (30 ml). The suspensions were stirred (150 rpm, 15 min) and centrifuged (2,800 ×*g*, 4^o^C, 20 min), and the supernatants were filtered through grade 1 filter paper **(**Whatman^®^ **).** The filtrates were extracted three times with diethyl ether **(**60 ml**)**, and the aqueous extract was collected and adjusted to pH 1.5 by 12 M HCl. The acidic aqueous solution was then filtered through grade 1 filter paper **(**Whatman^®^ **)** and extracted three times with diethyl ether **(**60 ml**).** The diethyl ether extract was collected, dried over Na_2_SO_4_ **(**anhydrous**)**, and filtered through grade 1 filter paper **(**Whatman^®^ **).** The filtrate was evaporated by a rotary evaporator, and subsequently blown with N_2_ gas. The extract was then dissolved in 50**%** methanol solution **(**1 ml**)** and filtered through a 0.22-µm filter (Minisart^®^). The phenolic compounds were analyzed by HPLC with diode-array detection (DAD) (Agilent Technologies, Germany) at 360 nm. Rutin (cat# R5143-50G, Sigma) and quercetin (cat# Q4951-10G, Sigma) were used as standards.

The HPLC system consisted of Waters in-line degasser DGU-20A5, SIL-20AHT autosampler, and SPD-M20A DAD. Empower software was used for data acquisition. The Waters system column C_18_ (4.6 × 250 mm, 5 µm particle diameter) coupled to a guard column was used. The column temperature was 25^o^C and the mobile phase flow rate was 2 ml/min. The phenolic compounds were eluted by a gradient elution of mobile phases A (100% acetonitrile) and B (50 mM potassium dihydrogen phosphate and 0.1% trifluoroacetic acid in deionized water), where A increased from 0% to 30% in 5 min, maintained at 30% for 2 min, and then resumed to 0% and maintained at 0% for 3 min before the next injection.

### 2.5 Evaluation of Non-cytotoxic Concentration of HC Extracts

#### 2.5.1 Evaluation in MARC-145 Cells

Filtered eluates of all HC extracts were serially diluted (2-fold) with MEM^++^, 100 µl of which was transferred into wells containing MARC-145 cells (100 µl; 10^4^ cells) in 96-well flat-bottom plates (Nunc, Denmark) in quadruplicate settings. The cultures were incubated for 96 h, then fixed with acetone:methanol solution. Subsequently, crystal violet (0.5%) and Sorenson’s buffer were added, and the O.D. values at 590 nm were determined. Untreated and vehicle controls were MARC-145 cells receiving MEM^++^ and vehicle solution, respectively. The O.D. values obtained from HC-treated or vehicle-treated cells were compared with those from untreated control and expressed as %viability of MARC-145 cells. The highest concentration of each HC extract that yielded O.D. values similar to those of untreated control was used for subsequent anti-PRRSV studies. The cytotoxicity tests were carried out in three independent studies.

#### 2.5.2 Evaluation in MDMs

Peripheral blood mononuclear cells (PBMCs) were isolated from whole blood by Ficoll-Hypaque gradient centrifugation (Histopaque^®^-1077, Sigma), and contaminating erythrocytes were eliminated as described previously (40). Greater than 95% viable PBMCs were obtained as determined by trypan blue staining. They were resuspended in RPMI^++^ prior to seeding (200 µl; 10^6^ cells) into 96-well flat-bottom plates in duplicate settings, and were cultured for 4 h. Non-adherent cells were removed and adherent cells were washed twice with pre-warmed (37^o^C) RPMI^++^ (200 µl) to obtain monocytes (4). The cells were then cultured in pre-warmed (37^o^C) RPMI^++^ (200 µl) for 48 h to obtain MDMs (4). Subsequently, the cultures received serially diluted (2-fold) filtered eluates of HC extracts (50 µl) and were incubated for 36 h prior to determination of cytotoxicity by trypan blue staining. Untreated and vehicle controls were MDMs receiving RPMI^++^ and vehicle solution, respectively. The cytotoxic assays were carried out in three independent studies. The highest concentration of each HC extract that showed no cytotoxic effects to MDMs, compared to untreated control, was used for subsequent studies.

### 2.6 Optimization of Incubation Period for Immune-Related Gene Expressions in MDMs Treated With HC Extracts

MDMs (in 200 µl of RPMI^++^) received filtered eluates of HC extracts (50 µl) at their non-cytotoxic concentration in duplicate settings. The cultures were incubated for 12, 24, and 36 h, then harvested and subjected to real-time PCR. Untreated and vehicle controls were MDMs receiving RPMI^++^ and vehicle solution, respectively. Positive control was MDMs stimulated with either polyinosinic:polycytidylic acid (poly IC, 5 μg/ml final; Sigma) or LPS from *Escherichia coli* O111:B4 (5 μg/ml final; Sigma). Poly IC was used as an inducer for gene expressions of myxovirus resistance 1 (*Mx1*), *IRF3*, *IRF7*, 2′-5′-oligoadenylatesynthetase 1 (*OAS1*), stimulator of interferon genes (*STING*), osteopontin (*OPN*), *IFNα*, *IFNβ*, *IL-10*, and *TGFβ*, while LPS was used as an inducer for gene expressions of *IFNγ* and *TNFα* (19).

### 2.7 Evaluation of Anti-PRRSV Activity of HC Extracts

#### 2.7.1 Anti-PRRSV Infectivity

Filtered eluates of all HC extracts (50 µl) or vehicle solution were incubated for 1 h with 100 µl of 10-fold serially diluted PRRSV-1 or HP-PRRSV-2 starting at 10^4^ TCID_50_ [equivalent to a multiplicity of infection (m.o.i.) of 1]. The mixtures were subsequently transferred to wells containing MARC-145 cells (100 µl; 10^4^ cells). The cultures were incubated for 1 h, then the supernatant was removed, and the cells were washed and received pre-warmed (37^o^C) MEM^++^ (250 µl). The cultures were incubated further for 96 h prior to IPMA. MARC-145 cells that received only serially diluted viruses served as virus control. The assays were carried out in three independent studies.

#### 2.7.2 Anti-PRRSV Replication

Tenfold serially diluted PRRSV-1 or HP-PRRSV-2 (100 µl; starting at m.o.i. of 1) was pipetted into wells containing MARC-145 cells (100 µl; 10^4^ cells) and the cultures were incubated for 1 h. Then, the supernatant was removed, and the cells were washed and received pre-warmed (37^o^C) MEM^++^ (200 µl) and 50 µl of either filtered eluate of HC extract or vehicle solution. The cultures were incubated further for 96 h prior to IPMA. MARC-145 cells that received only serially diluted viruses served as virus control. The assays were conducted in three independent studies.

### 2.8 Evaluation of Immunomodulatory Effects of HC Extracts on Immune-Related Gene Expressions in HP-PRRSV-2-Inoculated MDMs

MDMs (in 200 µl RPMI^++^) received either HP-PRRSV-2 (100 µl; 10^4^ TCID_50_) or mock Ags (100 µl) in duplicate settings. The cultures were incubated for 48 h prior to determination of cell viability by trypan blue staining. Subsequently, supernatant (100 µl) was gently removed, and MDMs that were incubated with HP-PRRSV-2 then received 50 µl of either RPMI^++^ (referred to as HP-PRRSV-2-inoculated) or filtered eluate of HC extract (referred to as HP-PRRSV-2-inoculated/HC-treated). MDMs that were incubated with mock Ags then received 50 µl of either RPMI^++^ (referred to as mock control) or vehicle solution (referred to as vehicle control). The cultures were incubated for 12 h, then harvested, determined for cell viability by trypan blue staining, and subjected to real-time PCR.

### 2.9 Real-Time PCR for Immune-Related Gene Expressions

Isolation of total RNA and elimination of contaminating DNA were carried out using NucleoSpin^®^ Blood kit (Macherey-Nagel, Bethlehem, PA). Complete elimination of contaminating DNA was confirmed by real-time PCR using total RNA and primers for two reference genes, i.e., *RPL32* (ribosomal protein L32) and *YWHAZ* (tyrosine 3-monooxygenase/tryptophan 5-monooxygenase activation protein, zeta). RNA concentration (A260) and purity (A260/280 and A260/230) were determined by spectrophotometry (NanoDrop 1000, NanoDrop Technologies, Montchamin, DE). All RNA samples had A260/280 and A260/230 between 2.0 and 2.2 and between 1.8 and 2.2, respectively. Integrity of RNA was determined by denaturing agarose gel electrophoresis followed by ethidium bromide staining. Reverse transcription was conducted, using RevertAid^TM^ First Strand cDNA synthesis kit (Thermo Fisher Scientific Baltics, Lithuania). The reaction used 500 ng of total RNA as template, and a mixture of oligo-dT and random hexamers as primers. cDNA was stored at −20°C until real-time PCR.

Real-time PCR was carried out in a 20-µl volume, consisting of 2 µl of cDNA, 10 μl of SYBR^®^ Green master mix (Toyobo, Japan), and optimized concentrations of primers (19). All reactions were set up in duplicate. The PCR condition was 95^o^C (15 min), and 40 cycles of 94^o^C (15 s), optimized annealing temperature (30 s) (19), and 72^o^C (30 s). The 2^−ΔΔCT^ method was used to evaluate immune-related gene expressions. The expressions of immune-related genes were normalized to the geometric average of *RPL32* and *YWHAZ* of the same animal and calibrated to that in the mock controls. The expression levels of immune-related genes were transformed into log2 scale. Melting curve analysis and agarose gel electrophoresis were conducted to verify a single product. In each run, nuclease-free water was included as no template control.

### 2.10 *In vivo* Experiment

Pigs were divided into 4 groups of 6 pigs (3 castrated male and 3 female). Group 1 was administered i.m. at the left side of the neck (needle 21G, 1” long) with 2 ml of Amervac^®^ PRRS MLV (Batch 97PT-6; Hipra) at 0 dpv (day post vaccination). Group 2 was administered i.m. (same condition as group 1) with 2 ml of Amervac^®^ PRRS MLV at 0 dpv and orally with HC50 extract (100 mg/ml; 2 ml/pig/day) *via* syringe at 0–49 dpv. The 200 mg/pig/day of HC extract was considered a safe dose for weaning pigs and did not have a negative effect on palatability when fed orally (41). Group 3 was administered i.m. (same condition as group 1) with 2 ml of vaccine solvent (Batch 4C79-1; Hipra) used for resuspension of lyophilized Amervac^®^ PRRS MLV at 0 dpv (served as challenge control group). Group 4 received no injection (served as strict control group). All groups except group 2 were administered orally with vehicle solution as placebo. All groups except group 4 were challenged i.n. with 1 ml of 10^5^ TCID_50_ of HP-PRRSV-2 at 28 dpv [equivalent to 0 dpc (day post challenge)]. Blood was collected weekly from the jugular vein at 0–49 dpv. Experimental schedule is summarized in **Table 1**. All serum samples were aliquoted and stored at −20^o^C until analysis for PRRSV-specific antibody (Ab) or at −70^o^C until analysis for the presence of PRRSV.

**Table 1:** Summary of the *in vivo* experiment.

Group	*N*	Treatment	Vaccine(dpv^*^)	HC50(dpv)	Challenge(dpv)	Blood collection (dpv)	Rectal temperature/Clinical score(dpv)	Body weight(dpv)
1	6	MLV	0	–	28	0, 7, 14, 21, 28, 35, 42, 49	28–49	0, 28, 49
2	6	MLV+HC50	0	0–49	28	0, 7, 14, 21, 28, 35, 42, 49	28–49	0, 28, 49
3	6	Vaccine solvent	0	–	28	0, 7, 14, 21, 28, 35, 42, 49	28–49	0, 28, 49
4	6	None	–	–	–	0, 7, 14, 21, 28, 35, 42, 49	28–49	0, 28, 49

^*^dpv, day post vaccination.

### 2.11 Clinical Scores and Growth Performance

Clinical scores (**Table 2**) (37) and rectal temperature were monitored daily at 0–21 dpc. Weight gain (WG) was calculated prior to (0–28 dpv) and after the HP-PRRSV-2 challenge (0–21 dpc). Average daily weight gain (ADWG) was calculated after the HP-PRRSV-2 challenge (0–21 dpc).

**Table 2:** Criteria for clinical scoring following the HP-PRRSV-2 challenge.

Parameters	Score			
	0	1	2	3
Respiration	Normal (<40/min)	Tachypnea (40–60/min)	Tachypnea (60–80/min)+Dyspnea	Tachypnea (>80/min) labored breathing
Behavior	Normal	Apathetic but responding to stimulation	Apathetic even when stimulated but still stand up	Prostration
Appetite	Normal	Anorexic	No	–
Discoloration of the ears	Normal	Red/Pale/Gray/Yellow	Purple/Blue	Ear necrosis
Other (conjunctivitis, coughing, sneezing, vomiting, diarrhea, shivering)	None	One sign presented	Two signs presented	Three or more signs presented

### 2.12 Ab Response

PRRSV-specific total and neutralizing Ab (nAb) were determined by ELISA (IDEXX PRRS X3 Ab Test, IDEXX Laboratories) and serum virus neutralization (SVN) test, respectively (28). HP-PRRSV-2 (10^2^ TCID_50_) was used as an *in vitro* Ag for SVN test. The cutoff titer for SVN test was at 2^2^.

### 2.13 Immune-Related Gene Expressions in Recall Reactions of PBMCs *ex vivo*

PBMCs (200 µl; 10^5^ cells) were cultured with HP-PRRSV-2 (100 µl; 10^4^ TCID_50_) or mock Ags (100 µl) for 72 h in duplicate. Total RNA was isolated and real-time PCR was carried out as described above.

### 2.14 Viremia

#### 2.14.1 Real-Time PCR for Viremic PRRSV

Isolation of viral RNA was conducted from 150 μl of serum samples, using Nucleospin^®^ RNA virus kit (Macherey-Nagel). Elimination of contaminating DNA was performed using rDNase (Macherey-Nagel). Complete elimination of contaminating DNA was confirmed by real-time PCR using total RNA and primers for *RPL32* and *YWHAZ*. All RNA samples had A260/280 and A260/230 between 2.0 and 2.2 and between 1.8 and 2.2, respectively. Reverse transcription was conducted using the RevertAid^TM^ First Strand cDNA synthesis kit.

Real-time PCR was carried out in a 20-µl volume, consisting of 2 µl of cDNA, 10 µl of SYBR^®^ Green PCR master mix (Toyobo), and 400 nM each of primer ORF7 149F and ORF7 346R (28). All reactions were set up in duplicate. The PCR condition was 95^o^C (15 min), and 35 cycles of 95^o^C (15 s), 53^o^C (30 s), and 72^o^C (30 s) (28). Recombinant PRRSV ORF7 plasmids (10^1^–10^8^ copy numbers) were used to generate a C_T_ standard curve (28). Melting curve analysis and agarose gel electrophoresis were conducted to verify a single product. In each run, nuclease-free water was included as no template control.

#### 2.14.2 Virus Isolation

Diluted serum samples (1:5 in MEM^++^; 200 µl) were inoculated onto MARC-145 cells (100 µl; 10^4^ cells) in 96-well flat-bottom plates. The cultures were incubated for 7 days prior to IPMA (37).

### 2.15 Data Analysis

Statistical analyses were performed using the SPSS software version 17 (IBM, Armonk, NY). Mean differences of immune-related gene expressions between HC-treated MDMs and vehicle control were tested by Student’s *t*-test. Mean differences of virus titers, WG, and ADWG among groups were determined by one-way ANOVA, followed by Dunnett’s test. Mean differences of s/p ratios, immune-related gene expressions, viremic PRRSV copy numbers, rectal temperature, and clinical scores among groups of pigs were tested by one-way repeated measures ANOVA, followed by Dunnett’s test. Statistical difference in the number of viremic pigs was tested by Poisson regression analysis. *p* < 0.05 was set as a statistically significant level.

## 3 Results

### 3.1 HPLC Chromatograms of HC Extracts

**Figure 1:**
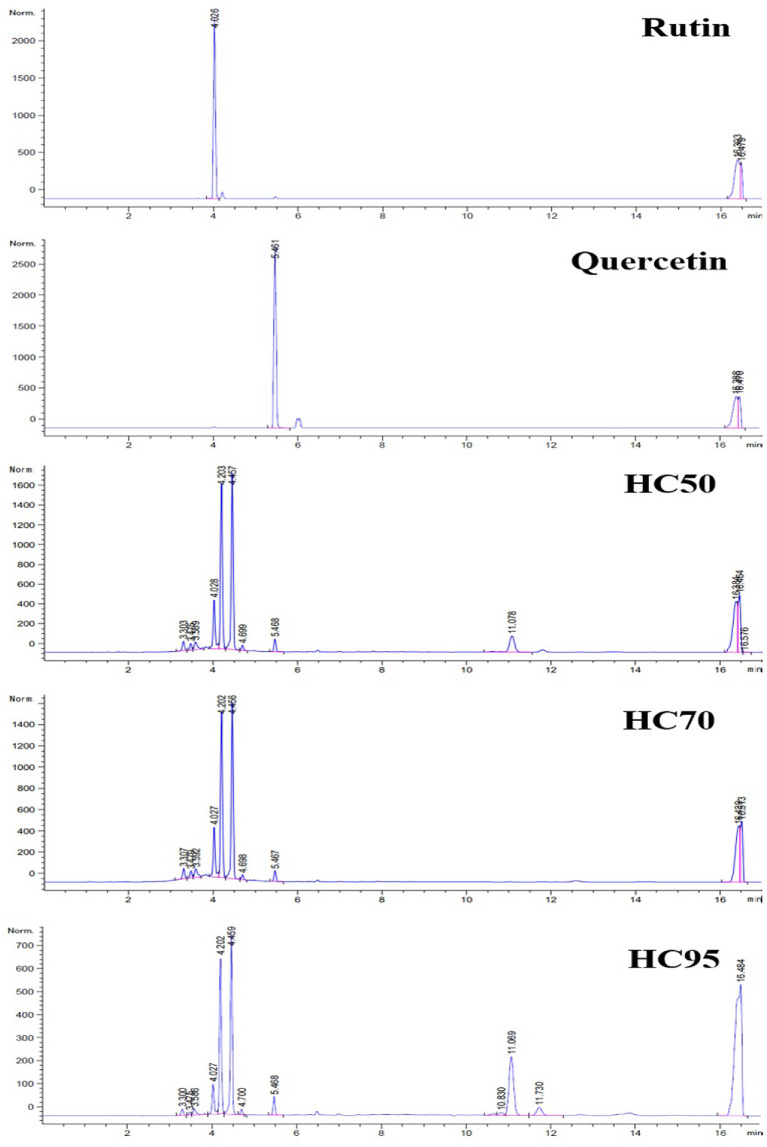
High-performance liquid chromatography (HPLC) chromatograms of rutin, quercetin, HC50, HC70, and HC95.

All HC extracts contained flavonoids, including rutin and quercetin (**Figure 1**). These were shown in chromatograms at the retention time (Rt) of 4.026 (rutin) and 5.461 (quercetin) min (**Figure 1**). There were at least two additional flavonoids that showed distinct peaks at Rt 4.202–4.203 and 4.456–4.459 min (**Figure 1**).

### 3.2 Optimal Concentrations of HC Extracts

**Figure 2:**
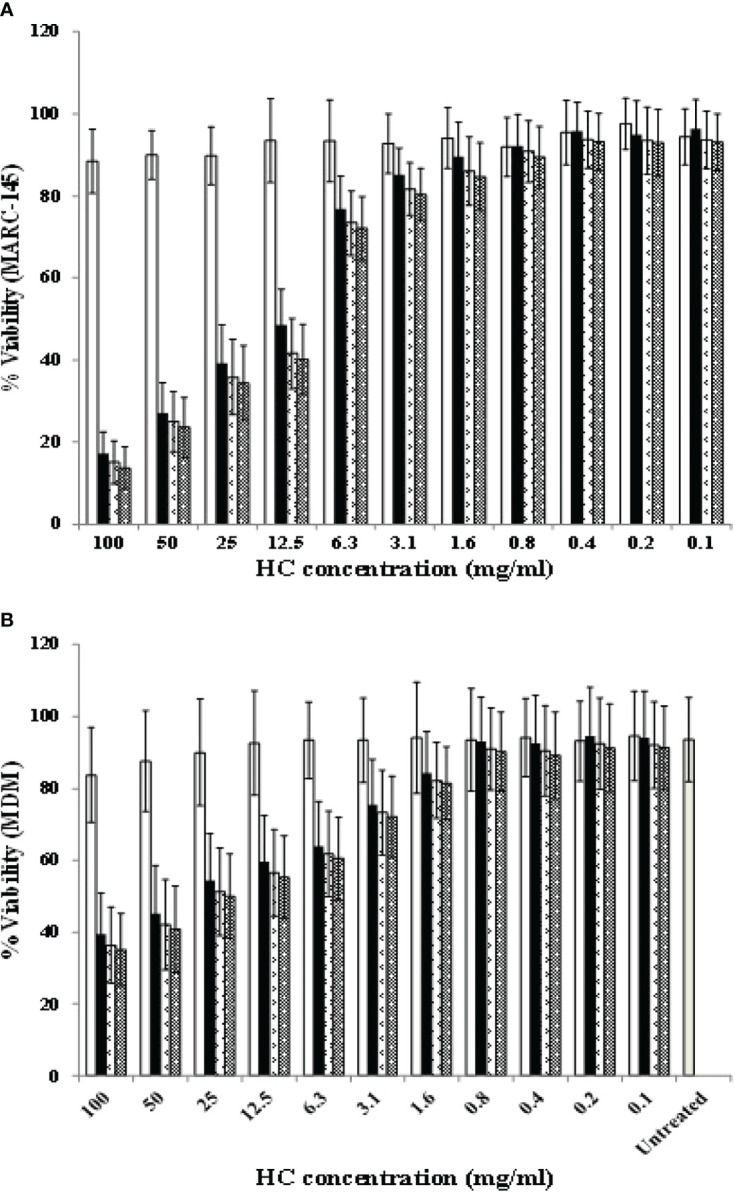
Determination of non-cytotoxic concentration of HC50, HC70, and HC95. **(A)** MARC-145 cells were incubated with 2-fold serially diluted HC50 (black bar), HC70 (divot bar), and HC95 (dotted bar), each starting at a final concentration of 100 mg/ml for 96 h. The cultures were then fixed with acetone:methanol solution, and stained with 0.5% crystal violet and Sorenson’s buffer. MARC-145 cells incubated with vehicle solution served as vehicle control (white bar). The O.D. values obtained from MARC-145 cells treated with HC50, HC70, HC95, and vehicle solution were compared with those from MARC-145 cells incubated with only MEM^++^ (untreated control) and expressed as %viability. **(B)** MDMs (*n* = 4 pigs) were incubated with 2-fold serially diluted HC50 (black bar), HC70 (divot bar), and HC95 (dotted bar), each starting at a final concentration of 100 mg/ml for 36 h. The cultures were then harvested and stained with trypan blue. MDMs incubated with vehicle solution served as vehicle control (white bar). Error bar indicates the standard deviation (SD).

The final concentration at 0.8 mg/ml was the highest non-cytotoxic concentration of all HC extracts for MARC-145 cells and MDMs. MARC-145 cells treated with HC50, HC70, and HC95 at 0.8 mg/ml yielded a viability of 92.24 ± 7.61% (mean ± standard deviation), 90.84 ± 7.53%, and 89.44 ± 7.48%, respectively, as compared to 91.96 ± 7.15% of MARC-145 cells treated with vehicle solution (**Figure 2A**). MDMs treated with HC50, HC70, and HC95 at 0.8 mg/ml yielded a viability of 93.12 ± 12.24%, 91.02 ± 11.37%, and 90.27 ± 10.94%, respectively, as compared to 93.50 ± 14.20% of MDMs treated with vehicle solution (**Figure 2B**). Untreated MDM control had a viability of 93.61 ± 11.66%.

### 3.3 Optimal Stimulation Period for Immune-Related Gene Expressions in MDMs Treated With HC Extracts

**Figure 3:**
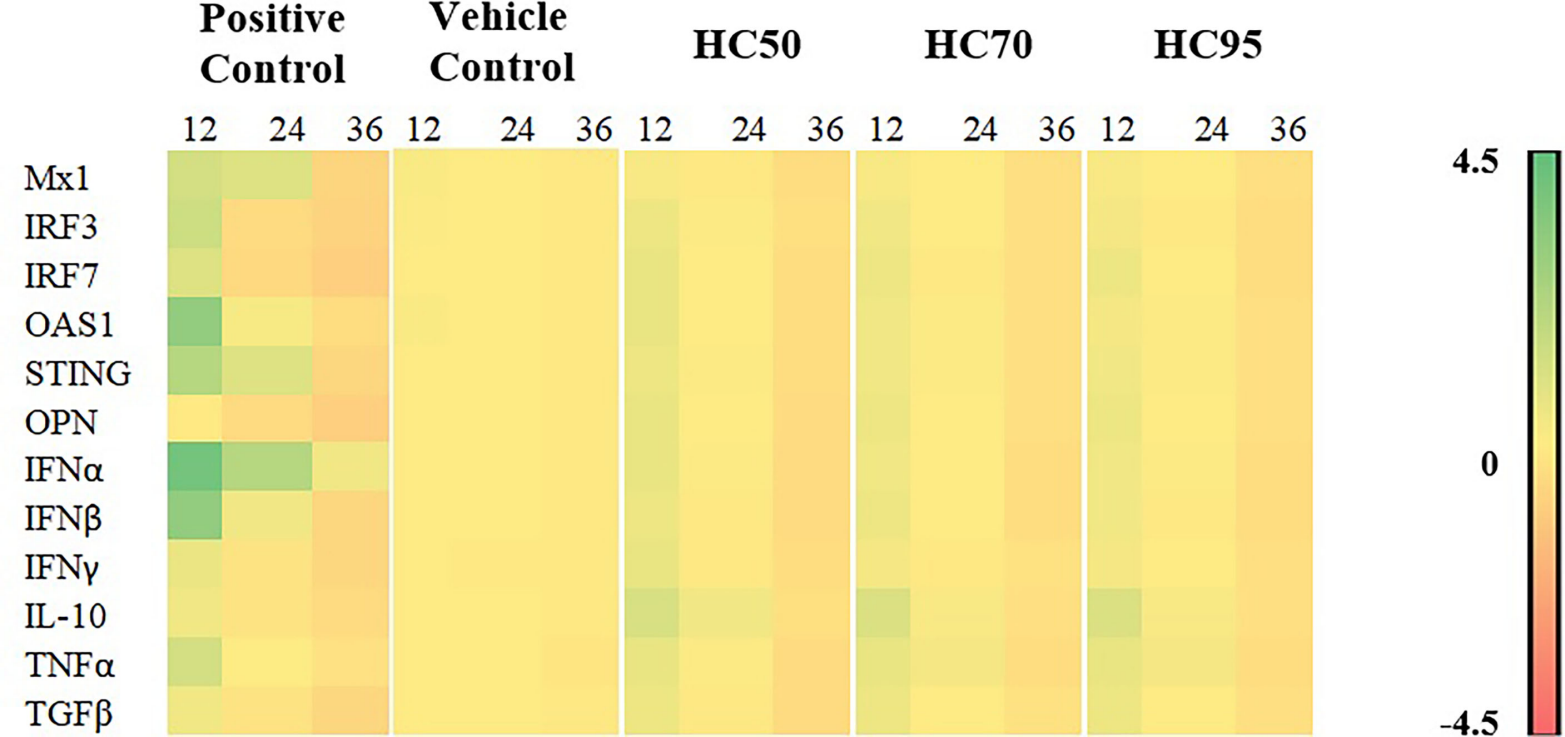
Heat map illustrating levels of immune-related gene expressions of MDMs (*n* = 4 pigs) treated with HC50, HC70, and HC95 (0.8 mg/ml final) for 12, 24, and 36 h as determined by real-time PCR. MDMs stimulated with poly IC (for induction of *Mx1*, *IRF3*, *IRF7*, *OAS1*, *STING*, *OPN*, *IFNα*, *IFNβ*, *IL-10*, and *TGFβ* gene expressions) or LPS (for induction of *IFNγ* and *TNFα* gene expressions) served as positive control. mRNA expressions of immune-related genes were normalized to geometric average of two housekeeping genes, i.e., *RPL32* and *YWHAZ*, of the same pigs. Expressions of immune-related genes in all groups were transformed to log2 scale and were presented in fold change, according to the 2^−ΔΔCT^ method, relative to those in untreated control.

Stimulation of MDMs with all HC extracts for 12 h resulted in the highest expression of all immune-related genes, compared with other incubation periods (**Figure 3** and **Supplementary Table 1**).Stimulation of MDMs with HC50, but not HC70 and HC95 extracts, still significantly induced expression of all immune-related genes after 24 h of incubation, compared with vehicle control. No stimulation of immune-related gene expression was detected in any HC extract at 36 h of incubation.

### 3.4 All HC Extracts Significantly Interfered With PRRSV Replication, but Not PRRSV Infectivity

**Figure 4:**
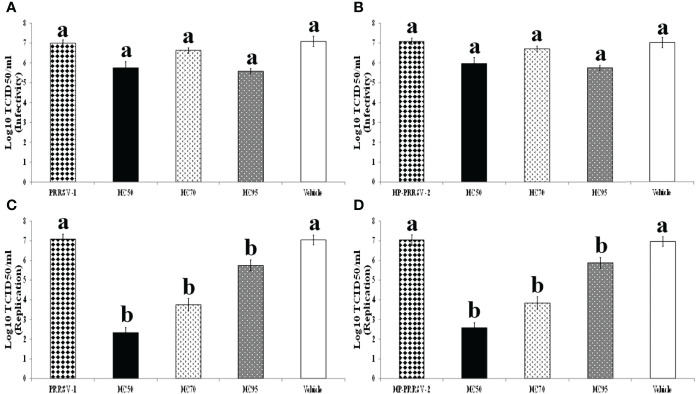
Effects of HC50, HC70, and HC95 on PRRSV-1 and HP-PRRSV-2 infectivity and replication *in vitro*. (Top panel) HC50 (black bar), HC70 (divot bar), and HC95 (dotted bar) were incubated with 10-fold serially diluted PRRSV-1 **(A)** and HP-PRRSV-2 **(B)** for 1 h prior to subsequent inoculation onto adherent MARC-145 cells. The cultures were incubated for 1 h, then the supernatants were removed, and the cells were washed and incubated further in MEM^++^ for 96 h. (Bottom panel) MARC-145 cells were inoculated with 10-fold serially diluted PRRSV-1 **(C)** and HP-PRRSV-2 **(D)** for 1 h, then the supernatants were discarded, and the cultures were washed and further incubated with HC50 (black bar), HC70 (divot bar), and HC95 (dotted bar) for 96 h. The virus titers were determined by IPMA. MARC-145 cells receiving only serially diluted PRRSV and vehicle solution served as PRRSV and vehicle controls, respectively. One-way ANOVA, followed by Dunnett’s test was used to determine the significant difference in mean virus titers. Error bar indicates the SEM. Different letters represent a significant mean difference among groups (*p* < 0.05).

Incubation of all HC extracts with PRRSV prior to subsequent inoculation to MARC-145 cells did not reduce PRRSV infectivity (**Figures 4A, B**). However, addition of all HC extracts into PRRSV-infected MARC-145 cells significantly interfered with virus replication. The titers of PRRSV-1 in MARC-145 cells treated with HC50, HC70, and HC95 extracts were reduced to 2.33 ± 0.26, 3.75 ± 0.32, and 5.75 ± 0.27, respectively as compared to 7.08 ± 0.21 of PRRSV-1 control (**Figure 4C**). The titers of HP-PRRSV-2 in MARC-145 cells treated with HC50, HC70, and HC95 extracts were reduced to 2.58 ± 0.21, 3.83 ± 0.33, and 5.88 ± 0.24, respectively, as compared to 7.04 ± 0.26 of HP-PRRSV-2 control (**Figure 4D**). No effect of vehicle solution on PRRSV replication was observed.

### 3.5 HC Extracts Significantly Improved mRNA Expressions of Most or All Immune-Related Genes Evaluated in HP-PRRSV-2-Inoculated MDMs

**Figure 5:**
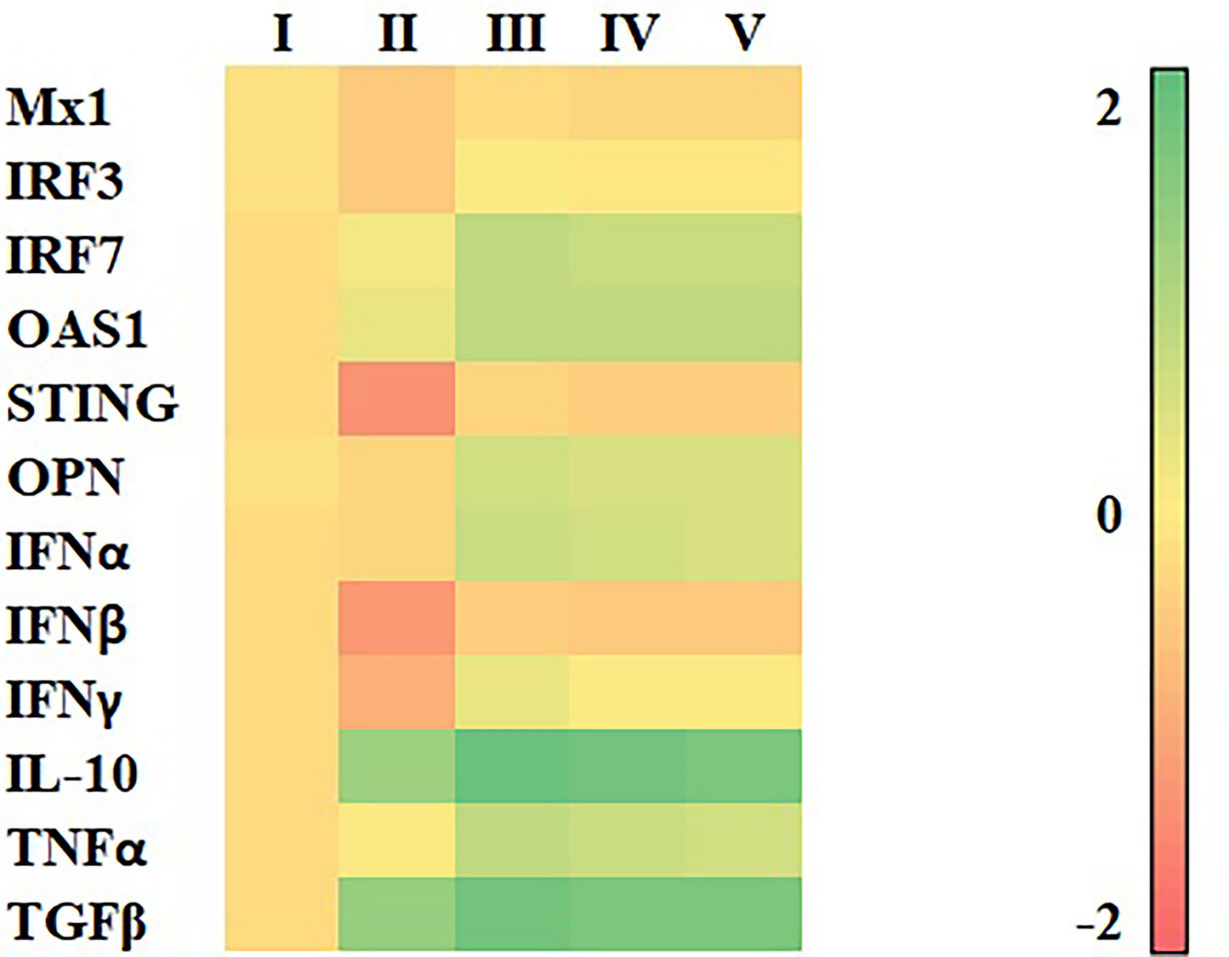
Heat map illustrating levels of immune-related gene expressions of MDMs (*n* = 4 pigs) inoculated with HP-PRRSV-2 and treated with either HC50, HC70, or HC95 (0.8 mg/ml final) as determined by real-time PCR. MDMs were inoculated with HP-PRRSV-2 for 48 h, then received either HC50, HC70, HC95, or RPMI^++^ for 12 h. Controls were MDMs incubated with mock Ags and received either vehicle solution (vehicle control) or RPMI^++^ (mock control). Levels of mRNA expressions of immune-related genes were normalized to geometric average of *RPL32* and *YWHAZ* of the same pigs. Expressions of immune-related genes in all groups were transformed to log2 scale and were presented in fold change, according to the 2^−ΔΔCT^ method, relative to those in mock control. I = Vehicle control; II = HP-PRRSV-2-inoculated MDMs; III = HP-PRRSV-2-inoculated/HC50-treated MDMs; IV = HP-PRRSV-2-inoculated/HC70-treated MDMs; and V = HP-PRRSV-2-inoculated/HC95-treated MDMs.

Compared with vehicle control, HP-PRRSV-2 significantly upregulated mRNA expressions of *IRF7*, *OAS1*, *IL-10*, *TNFα*, and *TGFβ*, and significantly downregulated mRNA expressions of *Mx1*, *IRF3*, *STING*, *IFNβ*, and *IFNγ* (**Figure 5** and **Supplementary Table 2**).

Compared with HP-PRRSV-2-inoculated MDMs, HP-PRRSV-2-inoculated/HC50-treated MDMs significantly upregulated mRNA expressions of all immune-related genes evaluated. HP-PRRSV-2-inoculated/HC70-treated MDMs also significantly upregulated mRNA expressions of all immune-related genes studied except *Mx1*. HP-PRRSV-2-inoculated/HC95-treated MDMs significantly upregulated mRNA expressions of all immune-related genes evaluated except *Mx1* and *IL-10*. The viability of MDMs at the time of gene expression analyses was greater than 70%.

### 3.6 Oral Supplementation of HC50 Extract Did Not Enhance PRRSV-Specific Ab Response Compared With PRRSV-1 MLV Vaccination Alone

**Figure 6:**
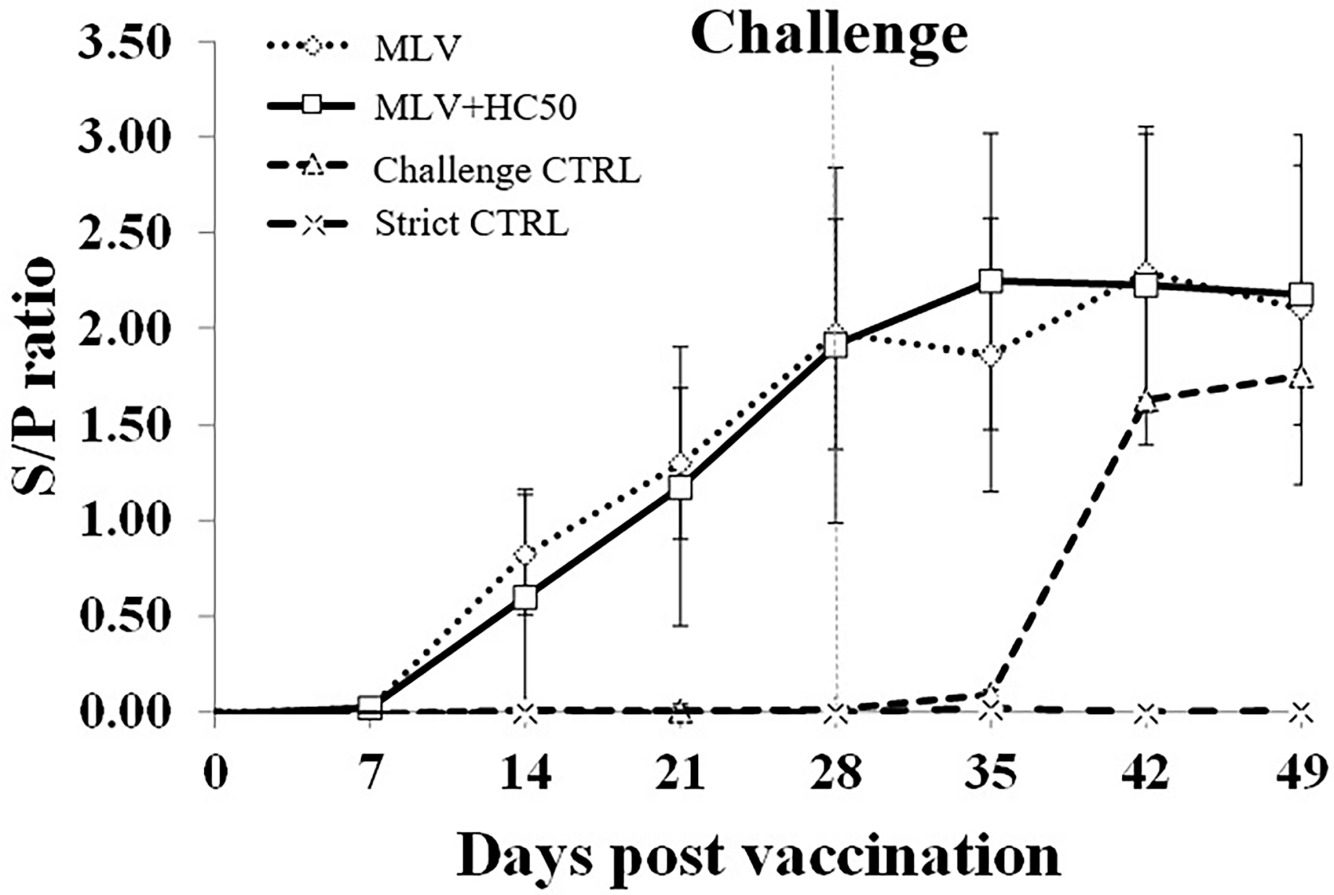
PRRSV-specific Ab response tested over time by ELISA. Pigs (*n* = 6) were injected i.m. with Amervac^®^ PRRS MLV (0 dpv) (MLV) and Amervac^®^ PRRS MLV (0 dpv) and fed p.o. with HC50 (0–49 dpv) (MLV+HC50) or vaccine solvent used for resuspension of lyophilized Amervac^®^ PRRS MLV (0 dpv) (Challenge CTRL). The animals were challenged i.n. with HP-PRRSV-2 at 28 dpv (dotted vertical line). Strict control pigs (Strict CTRL) received no treatment. Sera were collected weekly from all pigs at 0–49 dpv. The positive Ab response was determined at an s/p ratio of 0.4 or higher. One-way repeated measures ANOVA, followed by Dunnett’s test was used to determine the significant difference in mean s/p ratios (*p* < 0.05). Error bar indicates the SD.

Since HC50 extract showed more potent anti-PRRSV and immunomodulatory activities than HC70 and HC95 extracts, it was chosen for subsequent *in vivo* study. Both group 1 and group 2 had detectable PRRSV-specific Ab at 14 dpv, which peaked at 28 dpv (**Figure 6**). No anamnestic Ab response was seen in either group after the HP-PRRSV-2 challenge. No significant difference in s/p ratio was detected between group 1 and group 2 throughout the experiment. Group 3 had detectable PRRSV-specific Ab at 42 dpv (14 dpc). Group 4 remained PRRSV-seronegative throughout the experiment. No sex-related differences of PRRSV-specific Ab response and other immune-related parameters was detected. None of the groups developed nAb either prior to or after the HP-PRRSV-2 challenge (data not shown).

### 3.7 Immune-Related Gene Expressions *in vivo*

#### 3.7.1 Oral Supplementation of HC50 Extract Significantly Enhanced *IRF3* mRNA Expression in PRRSV-1 MLV-Vaccinated Pigs

**Figure 7:**
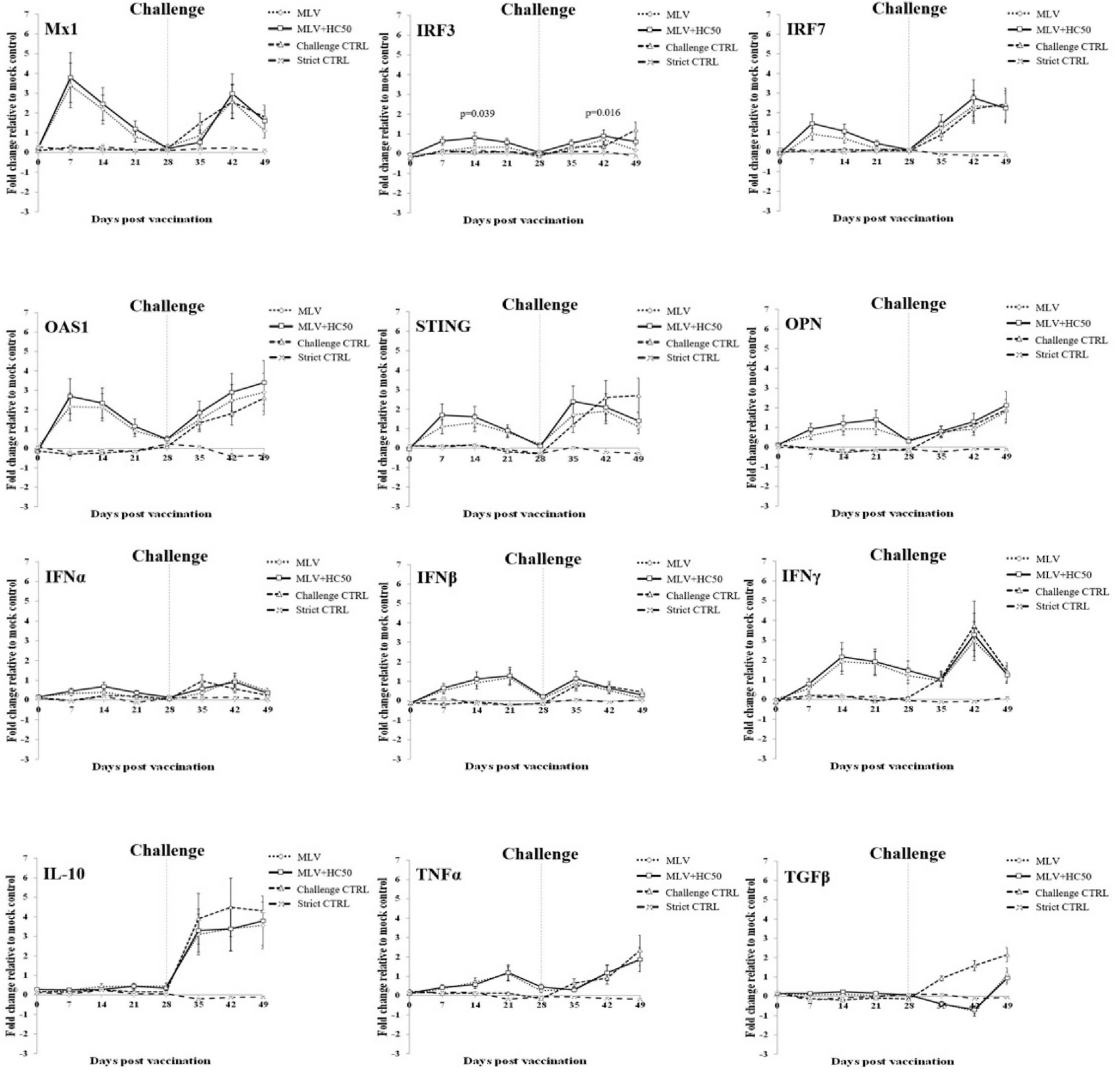
Immune-related gene expressions in PBMCs re-stimulated *ex vivo* with HP-PRRSV-2. Pigs (*n* = 6) were injected i.m. with Amervac^®^ PRRS MLV (0 dpv) (MLV) and Amervac^®^ PRRS MLV (0 dpv) and fed p.o. with HC50 (0–49 dpv) (MLV+HC50) or vaccine solvent used for resuspension of lyophilized Amervac^®^ PRRS MLV (0 dpv) (Challenge CTRL). The animals were challenged i.n. with HP-PRRSV-2 at 28 dpv (dotted vertical line). Strict control pigs (Strict CTRL) received no treatment. PBMCs were collected weekly from all pigs at 0–49 dpv. Harvested PBMCs were incubated with HP-PRRSV-2 for 72 h prior to determination of immune-related gene expressions by real-time PCR. PBMCs receiving mock Ag served as mock control. PBMCs stimulated with poly IC (for induction of *Mx1*, *IRF3*, *IRF7*, *OAS1*, *STING*, *OPN*, *IFNα*, *IFNβ*, *IL-10*, and *TGFβ* gene expressions) or LPS (for induction of *IFNγ* and *TNFα* gene expressions) served as positive control. mRNA expressions of immune-related genes were normalized to a geometric average of two housekeeping genes, i.e., *RPL32* and *YWHAZ*, of the same pigs. Expressions of immune-related genes in all groups were transformed to log2 scale and were presented in fold change, according to the 2^−ΔΔCT^ method, relative to those in mock control. One-way repeated measures ANOVA, followed by Dunnett’s test, was used to determine the significant difference in mean fold expressions (*p* < 0.05). Error bar indicates the SD. *p*-values of immune-related genes that showed significant difference between MLV and MLV+HC50 groups either prior to and after the HP-PRRSV-2 challenge was presented.

Following PRRSV-1 MLV vaccination, group 1 demonstrated significantly increased mRNA expressions of *IFNα* at 7 dpv, *IRF7* at 7–14 dpv, *IRF3* at 14–21 dpv, *IL-10* at 21–28 dpv, *Mx1* at 7–21 dpv, and *OAS1*, *STING*, *OPN*, *IFNβ*, *IFNγ*, *TNFα*, and *TGFβ* at 7–28 dpv, compared with group 3 (**Figure 7**).

Compared with group 1, group 2 showed significantly increased *IRF3* mRNA expression at 7–14 dpv. No difference in mRNA expression of any other immune-related genes was detected.

#### 3.7.2 Oral Supplementation of HC50 Extract Significantly Enhanced *IRF3* mRNA Expression in PRRSV-1 MLV-Vaccinated/HP-PRRSV-2-Challenged Pigs

Following the HP-PRRSV-2 challenge, group 1 demonstrated significantly decreased mRNA expressions of *IRF3* and *STING* at 21 dpc and of *TGFβ* at 7–21 dpc, compared with group 3. No difference in mRNA expression of any other immune-related genes was detected.

Compared with group 1, group 2 showed significantly increased *IRF3* mRNA expression at 21 dpc. No difference in any other immune-related gene expression was observed.

### 3.8 Oral Supplementation of HC50 Extract Reduced HP-PRRSV-2 Viremia

**Figure 8:**
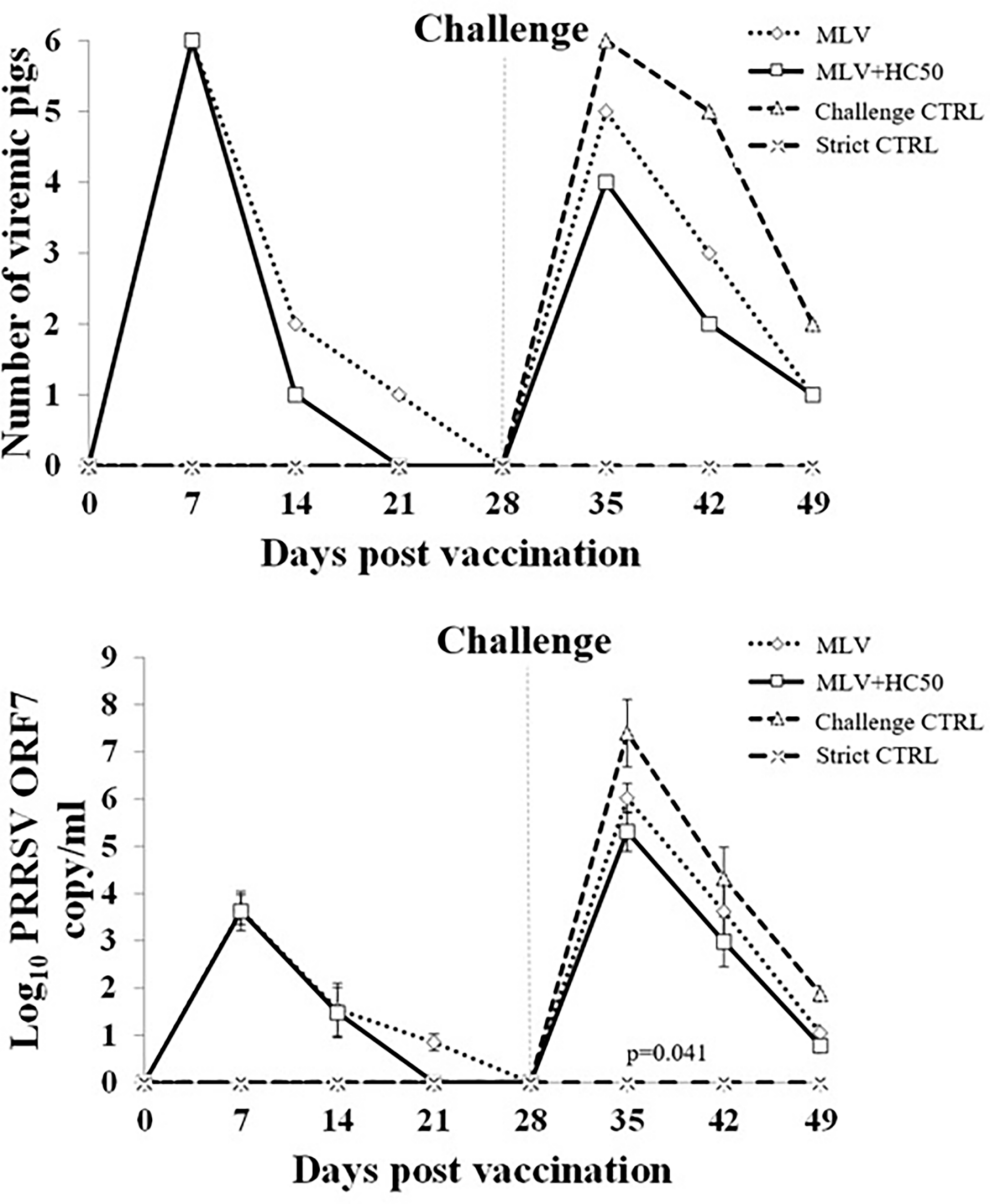
Number of viremic pigs and log_10_ PRRSV ORF7 copy numbers in serum following the HP-PRRSV-2 challenge. Pigs (*n* = 6) were injected i.m. with Amervac^®^ PRRS MLV (0 dpv) (MLV) and Amervac^®^ PRRS MLV (0 dpv) and fed p.o. with HC50 (0–49 dpv) (MLV+HC50) or vaccine solvent used for resuspension of lyophilized Amervac^®^ PRRS MLV (0 dpv) (Challenge CTRL). The animals were challenged i.n. with HP-PRRSV at 28 dpv (0 dpc) (dotted vertical line). Strict control pigs (Strict CTRL) received no treatment. Serum samples were collected weekly from all pigs at 0–21 dpc and were determined for PRRSV ORF7 copy numbers by real-time PCR. The C_T_ obtained from each sample was compared with the C_T_ standard curve generated from 10^1^ to 10^8^ copies of recombinant PRRSV ORF7 plasmids. The calculated copy numbers were transformed to log_10_ scale. Data were presented per ml of serum sample. Poisson regression analysis was used to determine the significant difference in the number of viremic pigs. One-way repeated measures ANOVA, followed by Dunnett’s test, was used to determine the significant difference in mean log_10_ PRRSV ORF7 copy numbers (*p* < 0.05). Error bar indicates the SD. *p*-value of statistical difference between MLV and MLV+HC50 groups after the HP-PRRSV challenge was presented.

Following PRRSV-1 MLV vaccination, group 1 and group 2 became viremic. PRRSV ORF7 gene and PRRSV was detected in 6/6, 2/6, 1/6, and 0/6 of group 1, and in 6/6, 1/6, 0/6, and 0/6 of group 2 at 7, 14, 21, and 28 dpv, respectively (**Figure 8**). No PRRSV ORF7 and PRRSV was detected in group 3 and group 4. The number of PRRSV ORF7 copies was significantly higher in group 1 than in group 2 at 21 dpv.

Following the HP-PRRSV-2 challenge, all groups except group 4 became viremic. PRRSV ORF7 gene and PRRSV was detected in 6/6, 5/6, and 2/6 of group 3 at 7, 14, and 21 dpc, respectively. The number of viremic pigs was decreased to 5/6, 3/6, and 1/6 of group 1, and to 4/6, 2/6, and 1/6 of group 2 at 7, 14, and 21 dpc, respectively. No significant difference in the number of viremic pigs was detected at any time point. The number of PRRSV ORF7 copies was significantly reduced in group 1 and group 2 as compared with that of group 3 (**Figure 8**). The number of PRRSV ORF7 copies was significantly reduced, by approximately 0.7 and 0.6 log_10_, in group 2 when compared to that of group 1 at 7 and 14 dpc.

### 3.9 Oral Supplementation of HC50 Extract Did Not Confer Clinical Protection or Improve Growth Performance

**Figure 9:**
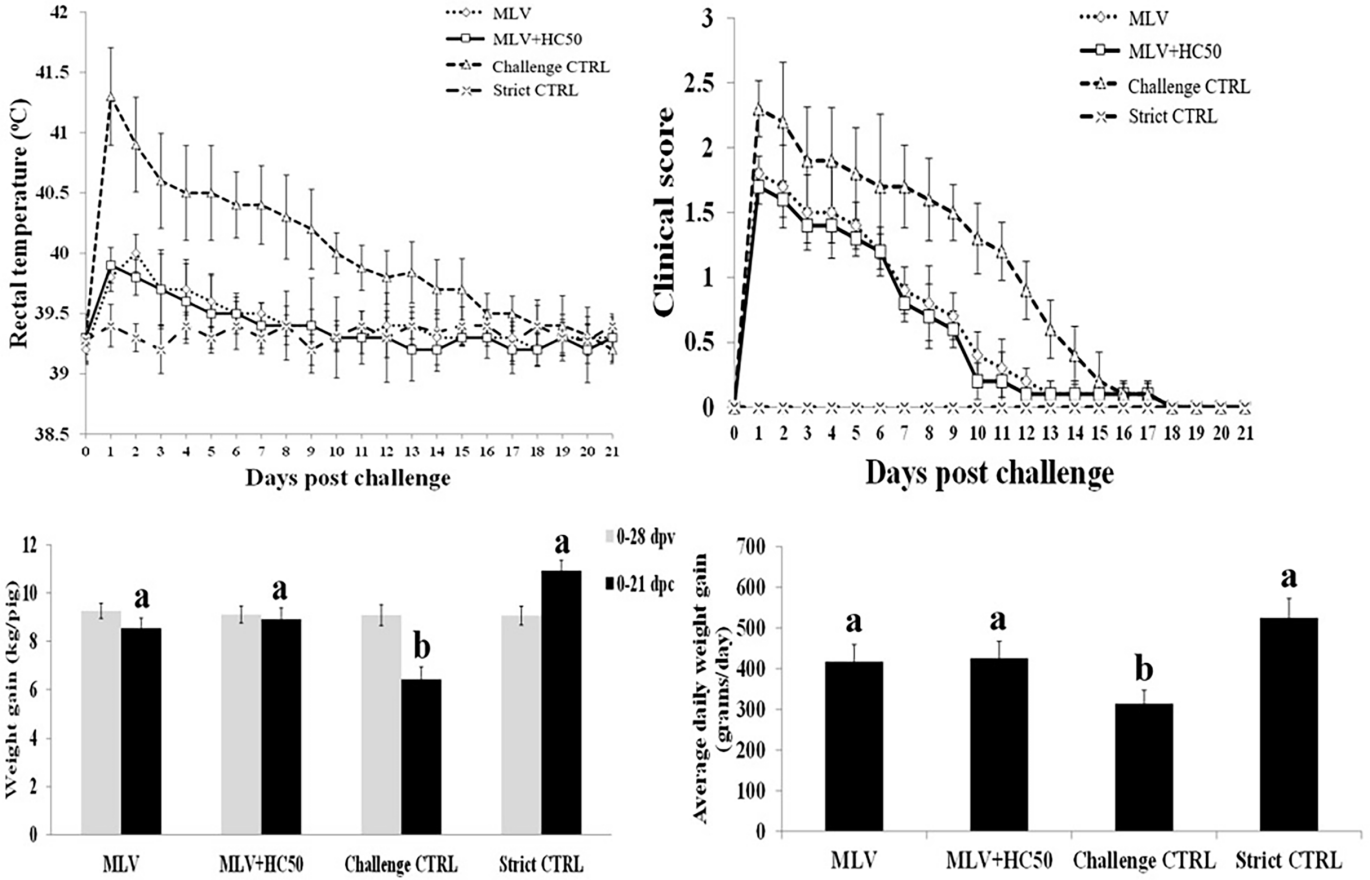
Rectal temperature, clinical score, weight gain (WG), and average daily weight gain (ADWG) of pigs following the HP-PRRSV-2 challenge. Pigs (*n* = 6) were injected i.m. with Amervac^®^ PRRS MLV (0 dpv) (MLV) and Amervac^®^ PRRS MLV (0 dpv) and fed p.o. with HC50 (0–49 dpv) (MLV+HC50) or vaccine solvent used for resuspension of lyophilized Amervac^®^ PRRS MLV (0 dpv) (Challenge CTRL). The animals were challenged i.n. with HP-PRRSV-2 at 28 dpv (0 dpc). Strict control pigs (Strict CTRL) received no treatment. Rectal temperature and clinical score were recorded daily. WG was calculated from 0 to 28 dpv, and from 0 to 21 dpc. ADWG was calculated from 0 to 21 dpc. One-way repeated measures ANOVA, followed by Dunnett’s test was used to determine the significant difference in mean rectal temperature and clinical score. One-way ANOVA, followed by Dunnett’s test, was used to determine the significant difference in mean WG and ADWG. Error bar indicates the SD. Different letters represent a significant mean difference among groups (*p* < 0.05).

Following the HP-PRRSV-2 challenge, group 3 developed high fever (rectal temperature above 40^o^C) from 1 to 10 dpc, which was gradually reduced to 39.5^o^C from 11 to 17 dpc (**Figure 9**). Group 1 and group 2 did not develop high fever and only had rectal temperature between 39.5 and 40^o^C from 1 to 7 dpc. Group 4 had rectal temperature under 39.5^o^C throughout the experiment. No significant difference in mean rectal temperature between group 1 and group 2 was detected at any time point.

Group 3 had higher clinical scores than group 1 and group 2 from 1 to 14 dpc (**Figure 9**). Group 1 and group 2 had clinical diseases from 1 to 10 dpc, with no significant difference in clinical scores between the two groups. Group 4 remained clinically normal throughout the experiment.

All groups had no difference in WG prior to the HP-PRRSV-2 challenge (**Figure 9**). Following the challenge, however, group 3 had significantly less WG and ADWG than group 1 and group 2. Group 3 had a WG of 6.42 ± 0.51 kg and an ADWG of 314.4 ± 33.1 g/day, while groups 1, 2, and 4 had a WG of 8.54 ± 0.42, 8.91 ± 0.47, and 10.93 ± 0.43, and an ADWG of 416.7 ± 42.2, 425.6 ± 41.2, and 525.1 ± 48.6 g/day, respectively (**Figure 9**).

## 4 Discussion

This study evaluated the *in vitro* anti-PRRSV activities of HC extracts in MARC-145 cells, the *ex vivo* immunomodulatory effects of HC extracts in HP-PRRSV-2-inoculated MDMs, and the *in vivo* effects of oral supplementation of HC50 extracts on improving the immunogenicity and cross-protective efficacy of the PRRSV-1 MLV vaccine against the HP-PRRSV-2 challenge.

All HC extracts contained rutin and quercetin as well as at least two other flavonoids (**Figure 1**). Both rutin and quercetin reportedly improved mRNA expressions of IRGs and type I and II IFN in porcine MDMs infected with HP-PRRSV-2 (37, 38). They also possessed anti-PRRSV infectivity and replicability in MARC-145 cells (37, 38). Other flavonoids that have been reportedly detected in HC ethanolic extracts include, for example, quercitrin, isoquercitrin, and houttuynoids (35, 36). Unlike rutin and quercetin, the immunomodulatory effects and anti-PRRSV activities of those flavonoids have not yet been studied in pigs in either *ex vivo* or *in vivo* settings.

All HC extracts were evaluated for their anti-PRRSV activities prior to studies of their immunomodulatory effects. The anti-PRRSV activities, if present, may not only be beneficial for PRRSV control, but also interfere with the expression levels of immune-related genes in HP-PRRSV-2-inoculated MDMs, and probably with the immunogenicity of the PRRSV-1 MLV vaccine. As shown in **Figures 4A, B**, treatment of PRRSV-1 and HP-PRRSV-2 with HC extracts prior to subsequent inoculation to MARC-145 cells did not reduce virus titers, suggesting that HC extracts do not possess virucidal activity. On the other hand, treatment of MARC-145 cells infected with PRRSV-1 or HP-PRRSV-2 with HC extracts significantly reduced virus titers by approximately 4.5 (HC50), 3 (HC70), and 1 (HC95) log_10_ TCID_50_/ml, respectively (**Figures 4C, D**). These findings suggest that HC50 and HC70 potentially reduce PRRSV replication. The significant reduction of PRRSV titers was not due to the cytotoxic effect of HC extracts and vehicle solution to MARC-145 cells (**Figure 2A**), and of vehicle solution to PRRSV (**Figures 4C, D**). The antiviral activities of HC extracts have been reported against some human and animal viruses, including herpes simplex virus (HSV) (35, 42–44), severe acute respiratory syndrome-coronavirus (SARS-CoV) (30), influenza A virus (IAV) (42, 45), human immunodeficiency virus type 1  (42), enterovirus 71 (46), infectious bronchitis virus (47), and mouse hepatitis virus (48). Some mechanisms of HC extracts for inhibition of these viruses include blocking of virus adsorption by binding to glycoproteins of HSV (35, 44), inhibition of NFκB activation and thereby reduced HSV replication (43), inhibition of 3C-like protease and RNA-dependent RNA polymerase activity of SARS-CoV (30), and inhibition of neuraminidase activity of IAV (45). The mechanisms of HC extracts against PRRSV replication are not known and need further studies.

All HC extracts significantly upregulated mRNA expressions of all immune-related genes evaluated in this study (**Figure 3** and **Supplementary Table 1**). Inoculation of HP-PRRSV-2 significantly upregulated mRNA expressions of two IRGs, i.e., *IRF7* and *OAS1*, and pro- and anti-inflammatory cytokines, i.e., *TNFα*, *IL-10*, and *TGFβ*, and significantly downregulated mRNA expressions of three IRGs, i.e., *Mx1*, *IRF3*, and *STING*, and *IFNβ* and *IFNγ* (**Figure 5** and **Supplementary Table 2**).The addition of HC50 extract to HP-PRRSV-2-inoculated MDMs significantly enhanced mRNA expressions of all immune-related genes, whereas the addition of HC70 and HC95 extracts significantly enhanced mRNA expressions of all immune-related genes except *Mx1* and *IL-10* (**Figure 5** and **Supplementary Table 2**). These findings suggest that HC extracts have the potential to upregulate mRNA expressions of IRGs, and type I and II IFN, which are downregulated by HP-PRRSV-2. It is essential to note that the modulation of immune-related gene expressions in HP-PRRSV-2-inoculated/HC-treated MDMs may need to take into account the anti-PRRSV activity of the HC extracts, although the incubation period of HC extracts in HP-PRRSV-2-inoculated MDMs was much shorter (12 h) than that in HP-PRRSV-2-inoculated MARC-145 cells (96 h). The difference in ORF7 copy numbers between HP-PRRSV-2-inoculated/HC-treated MDMs and HP-PRRSV-2-inoculated MDMs was not determined. It is noteworthy that, to our knowledge, this is the first study that reports the immunomodulatory effects of HC extracts on porcine immune cells.

Oral administration of HC50 extract was conducted at a daily dose of 200 mg/pig, which was considered a safe dose for weaning pigs (41). A previous study in growing pigs reported that oral administration of HC75 methanolic extract at a daily dose of approximately 350 mg/pig was safe and helped reduce *E. coli* concentration in the feces (41). The oral administration of HC50 extract neither improved nor reduced Ab response to the PRRSV-1 MLV vaccine, compared to group 1, which implied that the anti-PRRSV activity of HC50 extract did not interfere with PRRSV-1 MLV vaccine immunogenicity (**Figure 6**). The oral administration of HC50 extract, on the other hand, significantly enhanced *IRF3* expression at 7–14 dpv and 21 dpc, compared to group 1 (**Figure 7**).No difference in mRNA expressions of other immune-related genes was detected. Unlike *ex vivo* findings in MDMs, the immunomodulatory activities of HC50 extract on a significant upregulation of all immune-related gene expressions were not observed in recalled PBMCs. Further studies may consider a determination of additional immune parameters including an alteration of T-cell subpopulations and their immune-related activities to PRRSV.

Although oral administration of HC50 extract did not improve immunogenicity of the PRRSV-1 MLV vaccine, it helped reduce viremia (**Figure 8**). Prior to the HP-PRRSV-2 challenge, both group 1 and group 2 became viremic from PRRSV-1 MLV vaccination. Group 2 had a smaller number of viremic pigs at 14–21 dpv and significantly less copy numbers of PRRSV ORF7 at 21 dpv than group 1 (**Figure 8**). After the HP-PRRSV-2 challenge, group 2 had a smaller number of viremic pigs at 7–14 dpc, and significantly less copy numbers of PRRSV ORF7 at 7–14 dpc than group 1 (**Figure 8**). Fewer numbers of viremic pigs and PRRSV ORF7 copies in group 2 may be attributed to the anti-PRRSV activity of HC50 extract and probably the increased cross-protective efficacy of the PRRSV-1 MLV vaccine.

Apart from reduction of viremia, oral supplementation of HC50 extract did not protect pigs from fever and clinical diseases or improve growth performance as compared to group 1 (**Figure 9**). Both group 1 and group 2 had reduced degree and duration of fever and clinical diseases and had better growth performance than group 3, indicating a degree of cross-protection by the PRRSV-1 MLV vaccine against the HP-PRRSV-2 challenge (**Figure 9**). Different degrees of cross-protection of the PRRSV-1 MLV vaccine against PRRSV-2 challenge have been evidenced (28, 49, 50).

## 5 Conclusion

In conclusion, all HC extracts significantly reduced PRRSV replication in MARC-145 cells, and significantly enhanced IRGs, type I and II IFN, and pro- and anti-inflammatory cytokine expressions in HP-PRRSV-2-inoculated MDMs. Oral supplementation of HC50 extract significantly enhanced *IRF3* mRNA expression in recalled PBMCs, and reduced viremia in PRRSV-1 MLV-vaccinated/HP-PRRSV-2-challenged pigs.

## Data Availability Statement

The original contributions presented in the study are included in the article/**Supplementary Material**. Further inquiries can be directed to the corresponding author.

## Ethics Statement

The animal study was reviewed and approved by The Animal Care and Use Committee, Maejo University (Approval number MACUC 001S/2560).

## Author Contributions

WR was responsible for investigation, writing the original draft, and visualization. WC was in charge of conceptualization, methodology, validation, investigation, data curation, writing - review and editing, visualization, supervision, project administration, and funding acquisition. All authors reviewed the manuscript.

## Funding

This work is supported by the Royal Golden Jubilee Ph.D. program (grant # PHD/0132/2556).

## Conflict of Interest

The authors declare that the research was conducted in the absence of any commercial or financial relationships that could be construed as a potential conflict of interest.

## Publisher’s Note

All claims expressed in this article are solely those of the authors and do not necessarily represent those of their affiliated organizations, or those of the publisher, the editors and the reviewers. Any product that may be evaluated in this article, or claim that may be made by its manufacturer, is not guaranteed or endorsed by the publisher.
