# Supplementary material for: Oral Supplementation of Houttuynia cordata Extract Reduces Viremia in PRRSV-1 Modified-Live Virus-Vaccinated Pigs in Response to the HP-PRRSV-2 Challenge
Source: Front Immunol. 2022 Jul 18;13:929338. doi: 10.3389/fimmu.2022.929338 (PMC9339630; doi:10.3389/fimmu.2022.929338)
Supplement: Supplementary file 1 [file Table_1.docx]

Supplementary table 1 Immune-related gene expressions of MDMs (n=4 pigs) treated with HC50, HC70, and HC95 (0.8 mg/ml final) for 12, 24, and 36 h^*^.

| Gene | h | Pos Ctrl  ^**^ | Veh Ctrl | HC50 | HC70 | HC95 |
| --- | --- | --- | --- | --- | --- | --- |
| *Mx1* | 12 | 2.6+0.1 | 0.1+0.1 | 0.5+0.2^a^ | 0.4+0.1^a^ | 0.4+0.1^a^ |
|  | 24 | 2.4+0.1 | -0.1+0.1 | 0.2+0.1^a^ | 0.2+0.1^a^ | 0.2+0.1^a^ |
|  | 36 | 0.8+0.1 | -0.2+0.1 | -0.2+0.1 | -0.2+0.1 | -0.2+0.1 |
| *IRF3* | 12 | 2.7+0.1 | 0.0+0.1 | 0.8+0.1^a^ | 0.6+0.1^a^ | 0.5+0.1^a^ |
|  | 24 | 1.1+0.1 | -0.1+0.1 | 0.4+0.1^a^ | 0.2+0.1^a^ | 0.1+0.1 |
|  | 36 | 0.7+0.0 | -0.2+0.1 | -0.1+0.1 | -0.2+0.1 | -0.3+0.1 |
| *IRF7* | 12 | 2.4+0.1 | -0.1+0.0 | 0.9+0.2^a^ | 0.7+0.2^a^ | 0.7+0.1^a^ |
|  | 24 | 1.0+0.1 | -0.1+0.1 | 0.3+0.1^a^ | 0.1+0.1 | 0.2+0.1^a^ |
|  | 36 | 0.5+0.1 | -0.2+0.1 | -0.2+0.1 | -0.2+0.1 | -0.3+0.1 |
| *OAS1* | 12 | 3.7+0.1 | 0.1+0.1 | 0.9+0.1^a^ | 0.6+0.2^a^ | 0.5+0.2^a^ |
|  | 24 | 2.0+0.1 | -0.1+0.1 | 0.3+0.1^a^ | 0.3+0.1^a^ | 0.3+0.1^a^ |
|  | 36 | 1.2+0.1 | -0.2+0.1 | -0.2+0.1 | -0.2+0.1 | -0.2+0.1 |
| *STING* | 12 | 3.1+0.1 | -0.1+0.1 | 0.8+0.1^a^ | 0.6+0.1^a^ | 0.6+0.1^a^ |
|  | 24 | 2.4+0.1 | -0.1+0.1 | 0.4+0.1^a^ | 0.3+0.1^a^ | 0.3+0.2^a^ |
|  | 36 | 0.9+0.1 | -0.2+0.1 | -0.2+0.1 | -0.2+0.1 | -0.2+0.1 |
| *OPN* | 12 | 1.8+0.1 | -0.1+0.1 | 0.9+0.1^a^ | 0.7+0.1^a^ | 0.7+0.1^a^ |
|  | 24 | 1.1+0.1 | -0.1+0.1 | 0.3+0.2^a^ | 0.2+0.2 | 0.2+0.2 |
|  | 36 | 0.5+0.1 | -0.2+0.1 | -0.3+0.1 | -0.2+0.1 | -0.2+0.2 |
| *IFNα* | 12 | 4.2+0.1 | -0.1+0.1 | 0.9+0.1^a^ | 0.6+0.1^a^ | 0.6+0.1^a^ |
|  | 24 | 3.1+0.1 | -0.1+0.1 | 0.4+0.1^a^ | 0.2+0.1^a^ | 0.3+0.1^a^ |
|  | 36 | 2.1+0.1 | -0.2+0.1 | -0.3+0.1 | -0.3+0.1 | -0.3+0.1 |
| *IFNβ* | 12 | 3.7+0.1 | -0.1+0.1 | 0.8+0.2^a^ | 0.7+0.2^a^ | 0.6+0.1^a^ |
|  | 24 | 2.1+0.1 | -0.1+0.1 | 0.2+0.1^a^ | 0.2+0.1^a^ | 0.1+0.1 |
|  | 36 | 0.9+0.1 | -0.2+0.1 | -0.3+0.1 | -0.3+0.2 | -0.3+0.1 |
| *IFNγ* | 12 | 2.2+0.1 | -0.1+0.1 | 0.9+0.1^a^ | 0.5+0.2^a^ | 0.5+0.1^a^ |
|  | 24 | 1.5+0.1 | -0.2+0.1 | 0.2+0.1^a^ | 0.1+0.1^a^ | 0.2+0.1^a^ |
|  | 36 | 0.9+0.1 | -0.2+0.1 | -0.2+0.1 | -0.1+0.1 | -0.2+0.1 |
| *IL-10* | 12 | 2.1+0.1 | -0.1+0.1 | 1.4+0.2^a^ | 1.2+0.2^a^ | 1.2+0.2^a^ |
|  | 24 | 1.5+0.1 | -0.1+0.1 | 0.7+0.1^a^ | 0.4+0.1^a^ | 0.4+0.1^a^ |
|  | 36 | 1.1+0.1 | -0.2+0.1 | -0.1+0.1 | -0.2+0.1 | -0.2+0.1 |
| *TNFα* | 12 | 2.6+0.1 | -0.1+0.1 | 0.9+0.1^a^ | 0.8+0.2^a^ | 0.8+0.1^a^ |
|  | 24 | 1.9+0.1 | -0.1+0.1 | 0.4+0.1^a^ | 0.5+0.1^a^ | 0.5+0.1^a^ |
|  | 36 | 1.4+0.1 | -0.3+0.1 | -0.3+0.1 | -0.3+0.1 | -0.3+0.1 |
| *TGFβ* | 12 | 2.1+0.1 | -0.1+0.1 | 0.8+0.1^a^ | 0.7+0.2^a^ | 0.7+0.2^a^ |
|  | 24 | 1.5+0.1 | -0.1+0.1 | 0.2+0.1^a^ | 0.2+0.1^a^ | 0.2+0.1^a^ |
|  | 36 | 0.9+0.1 | -0.2+0.1 | -0.3+0.1 | -0.1+0.1 | -0.2+0.1 |

^*^Immune-related gene expressions are presented in log2 scale as mean fold change+standard deviation (SD), according to 2^(-ΔΔC_T_) method. The expressions were normalized to the geometric average of two housekeeping genes, i.e. *RPL32* and *YWHAZ* of the same pigs and were calibrated to those in untreated MDMs.

^**^Gene expressions in positive control (Pos Ctrl) were stimulated by Poly IC (for *Mx1*, *IRF3*, *IRF7*, *OAS1*, *STING*, *OPN*, *IFNα*, *IFNβ*, *IL-10*, and *TGFβ*) and LPS (for *IFNγ* and *TNFα*).

^a^indicates significant mean fold difference between vehicle control (Veh Ctrl) and MDMs treated with either HC50, HC70, or HC95 as determined by Student’s t test (p<0.05).

Supplementary table 2 Immune-related gene expressions of MDMs (n=4 pigs) inoculated with HP-PRRSV-2 and treated with either HC50, HC70, or HC95 (0.8 mg/ml final)^*^.

| Gene | Veh  Ctrl | HP-PRRSV-2-inoculated | HP-PRRSV-2-inoculated/ HC50-treated | HP-PRRSV-2-inoculated/ HC70-treated | HP-PRRSV-2-inoculated/  HC95-treated |
| --- | --- | --- | --- | --- | --- |
| *Mx1* | 0.0+0.1 | -0.4+0.1^a^ | -0.1+0.1^b^ | -0.2+0.1 | -0.2+0.1 |
| *IRF3* | 0.0+0.1 | -0.4+0.1^a^ | 0.2+0.1^b^ | 0.1+0.1^b^ | 0.1+0.1^b^ |
| *IRF7* | -0.1+0.1 | 0.3+0.1^a^ | 0.9+0.1^b^ | 0.8+0.1^b^ | 0.8+0.1^b^ |
| *OAS1* | -0.1+0.1 | 0.4+0.2^a^ | 0.9+0.1^b^ | 0.9+0.1^b^ | 0.9+0.2^b^ |
| *STING* | -0.1+0.1 | -1.3+0.2^a^ | -0.2+0.1^b^ | -0.3+0.1^b^ | -0.3+0.1^b^ |
| *OPN* | 0.0+0.1 | -0.2+0.1 | 0.7+0.1^b^ | 0.6+0.1^b^ | 0.6+0.1^b^ |
| *IFNα* | -0.1+0.1 | -0.2+0.1 | 0.8+0.1^b^ | 0.7+0.1^b^ | 0.6+0.1^b^ |
| *IFNβ* | -0.1+0.1 | -1.2+0.1^a^ | -0.3+0.1^b^ | -0.4+0.1^b^ | -0.4+0.1^b^ |
| *IFNγ* | -0.1+0.1 | -0.8+0.1^a^ | 0.4+0.1^b^ | 0.2+0.1^b^ | 0.2+0.1^b^ |
| *IL-10* | -0.1+0.1 | 1.3+0.2^a^ | 1.9+0.1^b^ | 1.8+0.2^b^ | 1.7+0.2 |
| *TNFα* | -0.1+0.1 | 0.2+0.1^a^ | 0.9+0.1^b^ | 0.8+0.1^b^ | 0.7+0.1^b^ |
| *TGFβ* | -0.1+0.1 | 1.4+0.1^a^ | 1.8+0.1^b^ | 1.7+0.1^b^ | 1.7+0.1^b^ |

^*^Immune-related gene expressions are presented in log2 scale as mean fold change+SD, according to 2^(-ΔΔC_T_) method. The expressions were normalized with mRNA expressions of two housekeeping genes, i.e. *RPL32* and *YWHAZ* of the same pigs and were calibrated to those in mock control.

^a^indicates significant mean fold difference between vehicle control (Veh Ctrl) and MDMs inoculated with HP-PRRSV-2 as determined by Student’s t test (p<0.05).

^b^indicates significant mean fold difference between HP-PRRSV-2-inoculated MDMs and HP-PRRSV-2-inoculated MDMs treated with either HC50, HC70, or HC95 as determined by Student’s t test (p<0.05).
